# A prediction method for older adult care service demand combining improved RF algorithm and logistic regression

**DOI:** 10.3389/fpubh.2025.1679228

**Published:** 2026-01-09

**Authors:** Ya Wang, Na Liu

**Affiliations:** Academy of Art and Design, Yancheng Teachers University, Yancheng, China

**Keywords:** feature selection, health of the older adult, logistic regression, nursing service demand, random forest

## Abstract

**Introduction:**

With the acceleration of global aging, the accurate prediction of care service demand for older adults is of significant importance for optimizing resource allocation. Traditional prediction methods often lack sufficient accuracy when dealing with high-dimensional and nonlinear health data.

**Methods:**

A prediction model integrating an improved random forest (RF) algorithm and logistic regression (LR) is proposed. The method introduces an adaptive feature selection strategy within the RF framework to dynamically select the most influential feature subsets. Key features screened by the optimized RF are then used to construct an LR classifier, leveraging its strengths in handling linear relationships and providing interpretability.

**Results:**

The proposed model achieved an accuracy of 95.30%, a recall rate of 92.60%, an F1 score of 93.90%, and an area under the receiver operating characteristic curve of 0.934 in predicting care service demand for older adults. These results were significantly better than those obtained using the RF or LR models alone.

**Discussion:**

The findings indicate that the integrated approach effectively improves prediction accuracy and reliability. The model offers a robust decision-support tool for care service institutions and government departments in resource planning and service allocation for the older population.

## 1 Introduction

The demand for older adult care services (DOACS) is growing as the global population continues to age. Accurately predicting their needs is crucial for optimizing resource allocation and improving service quality (1). Accurate demand forecasting can help older adult care service institutions and government departments better plan resources, ensuring that limited resources can be reasonably allocated to the older adult population who need them the most, thereby enhancing the efficiency and quality of the entire older adult care service system (2). However, the health data of the older adult usually has characteristics such as high dimensionality, non-linearity, and complexity. Traditional demand forecasting methods mainly rely on statistical analysis and simple machine learning (ML) models, which have problems of poor prediction accuracy (ACC) and low efficiency in predicting the nursing service needs of the older adult (3). In addition, existing methods have limitations in feature selection and model interpretability, making it difficult to comprehensively capture the key factors that affect nursing service demand (4). In recent years, Random Forest (RF) has become a popular ML algorithm for predictive modeling in the medical field, owing to its excellent feature processing and nonlinear modeling capabilities (5).

A. C. Hauschild et al. evaluated the performance of biomedical datasets using the federated RF model to address heterogeneity issues within and between datasets. The findings denoted that the model could integrate multi-source data without infringing on privacy, improve the model’s generalization ability, and facilitate precision medicine for patients with rare diseases and remote areas (6). Y. Xin and X. Ren proposed a prediction model based on RF classifier to identify the depression risk of older adult people with disabilities in urban and rural areas. This method predicted the early detection of depression in disabled older adult individuals by using RF data. The findings denoted that the average 10 fold cross validation results of the model in rural and urban areas were 0.71 and 0.70, respectively (7). S. Gundogdu developed an early diabetes prediction model by leveraging multiple linear regression and RF for feature selection, followed by classification with extreme gradient boosting. It was reported that the model achieved a prediction ACC of 99.2% and a response time of merely 0.048 s (8). M. G. El-Shafey et al. designed a hybrid GA and PSO method based on RF for predicting heart disease. This method used an improved GA for global search and PSO algorithm for local search. The findings denoted that the prediction ACC of this method reached 95.6 and 91.4% in two datasets, respectively, and was superior to existing methods (9). F. Mbonyinshuti et al. developed an RF-based method for predicting the demand for essential drugs to update and optimize the management of essential drugs used for treating diseases. The demand trend of various commonly used non-communicable disease essential drugs was predicted through consumption data of essential drugs. The outcomes denoted that the RF model had an ACC of 78% on the training set and 71% on the test set (10). D. H. Yang et al. designed a medical service volume prediction method based on ML algorithms such as RF. By integrating hospital, doctor, and patient features to construct a feature set, RF was used for feature selection, and offline medical indicators were introduced to analyze the impact relationship between online and offline. The findings denoted that the model had the highest ACC of 96.89% in predicting online consultation volume (11).

Logistic Regression (LR), as a classic classification algorithm, has good interpretability and the ability to handle linear relationships, and has been broadly utilized in the areas of medicine and health (12). R. Van den Goorbergh et al. established four prediction models using standard LR and penalty LR to address the issue of class imbalance, including uncorrected, random undersampling, and random oversampling. Different methods were compared through Monte Carlo simulation in terms of discrimination, calibration, and classification performance. The findings showed that the correction method enhanced the balance between sensitivity and specificity (13). G. Ambrish et al. developed a cardiovascular disease prediction method based on LR technology, which preprocesses the dataset, selects features highly positively correlated with the target value, and divides the dataset into different proportions for training and testing. The findings denoted that the LR model had the highest prediction ACC, at 87.10% (14). J. H. Oosterhoff et al. compared the effectiveness of ML and LR in predicting surgical outcomes in orthopedic trauma. By analyzing 9 musculoskeletal trauma datasets and developing a model using five fold cross validation, their performance was evaluated. The findings denoted that the average statistic of LR model was 0.01 higher than that of ML model, and there was a significant difference (15). A. Pate et al. used multiple LRs to develop a minimum sample size for multivariate prediction models and tested the effectiveness of the minimum model overfitting through simulation studies. The outcomes indicated that this method could control overfitting and improve the overall risk assessment effectiveness of the model (16). Z. S. Munmun et al. proposed an ML-based coronary heart disease classification and prediction method, which compared the predictive performance of LR, RF, and Support Vector Machines (SVMs). The model effectiveness was tested using indicators such as ACC, specificity, and sensitivity. The findings denoted that the ACC of RF was 93.5%, and the sensitivity of SVM was 97.5%, highlighting the potential of ML technology in clinical decision-making and personalized medicine (17).

Despite numerous scholars conducting research on RF algorithms and LR models in the fields of healthcare and wellness, significant achievements have been made. However, existing RF algorithms still suffer from redundant or irrelevant feature interference in the feature selection process, which affects the generalization performance of the model. In addition, although LR models perform well in handling linear relationships and have high interpretability, their predictive ability in complex data is limited. Therefore, combining the advantages of the two algorithms to build an efficient and accurate prediction model has become a current research focus. In view of this, research proposes a method for predicting the DOACS by integrating improved RF and LR, aiming to improve the ACC and efficiency of feature selection and achieve high-precision prediction of older adult care service demand. In this study, DOACS is defined as the need for formal care services, including but not limited to nursing care, rehabilitation, home health aid, and medical monitoring, as determined by professional assessment based on health and functional status. It is operationalized as a binary classification task: whether an individual requires significant care services or not, based on thresholds derived from expert guidelines and historical service usage.

The novelty of this study lies in: (1) Decomposing the original health signal into sub bands of different frequencies, thereby extracting multi-domain features such as time-domain (TD), frequency-domain (FD), and nonlinear-domain. Through multi-domain feature extraction (MDFE), key information in the data can be more comprehensively captured; (2) The dynamic feature selection mechanism based on adaptive selection threshold (AST) analyzes the distribution characteristics of feature importance and balances the screening strictness using adjustment coefficients to ensure that the feature dimension is controlled while retaining significantly relevant features, further optimizing the feature selection process; (3) Combining the Optimized Random Forest (ORF) algorithm with LR, fully leveraging the advantages of RF in nonlinear feature selection and LR’s features in handling linear relationships and providing interpretability.

## 2 Materials and methods

This section first proposes an RF optimization method grounded on discrete wavelet transform (DWT) MDFE to strengthen the model’s ability to handle high-dimensional and non-stationary data. Then, a prediction model combining optimized RF and LR is constructed to improve the ACC of predicting the DOACS.

### 2.1 RF optimization method based on MDFE using DWT

In predicting the DOACS, data often has high dimensionality, non-linearity, and complexity, and traditional feature extraction methods are difficult to effectively capture key information in the data. The RF algorithm is widely used due to its excellent nonlinear modeling ability and feature importance evaluation performance. However, traditional RF algorithms have shortcomings such as feature redundancy, noise interference, and insufficient MDFE when processing high-dimensional and non-stationary older adult health data (18). DWT, as a powerful signal processing tool, can decompose data into sub bands of different frequencies, thereby extracting multi-domain features including TD, FD, and energy-domain (19). Therefore, an MDFE method based on DWT is proposed, combined with adaptive feature selection strategy to optimize the feature processing capability of RF algorithm. This method utilizes DWT to perform time-frequency decomposition on the original health signal, extracting features in the field of view, FD, and nonlinear domain. By using the AST strategy, features with higher importance are retained. Finally, an RF model is constructed grounded on the filtered feature subset, and the classification performance is improved through a weighted voting mechanism.

In the context of this study, health signal refers to a structured, one-dimensional representation of an individual’s multivariate health profile. As the MEPS (20) and NHATS (21) datasets consist of tabular data, a signal was synthetically constructed for each participant by concatenating a selected set of static health variables into a fixed-order sequence. This approach allows for the application of signal processing techniques, such as DWT, to capture potential non-linear interactions and complex patterns within the combined feature set that might be overlooked by traditional methods. The initial variables considered for this construction were those related to core health and functional status, including: the number of chronic diseases, activities of daily living score, instrumental activities of daily living score, mental health score, total medical expenditures, frequency of hospitalizations, and medication count. Variables deemed non-informative for a holistic health status representation, such as direct identifiers and administrative codes, were excluded from the signal construction phase. The final feature vector for each individual was thus formed by the normalized values of these selected variables, arranged in a consistent order. This synthesized vector was subsequently subjected to DWT for multi-scale analysis and feature extraction in the time-frequency domain.

The study first decomposes the signal x(t) into DWT to obtain approximate coefficients and detail coefficients, to achieve time-frequency analysis of the original signal. The expression is shown in Equation 1 (22, 23).


$$\left\{ \begin{array}{l} cA_{j}[n]=\sum_{k}x[q]\cdot \xi_{j,n}[q]\\ cD_{j}[n]=\sum_{k}x[q]\cdot \varsigma_{j,n}[q] \end{array}\right.$$ (1)

**Figure 1:**
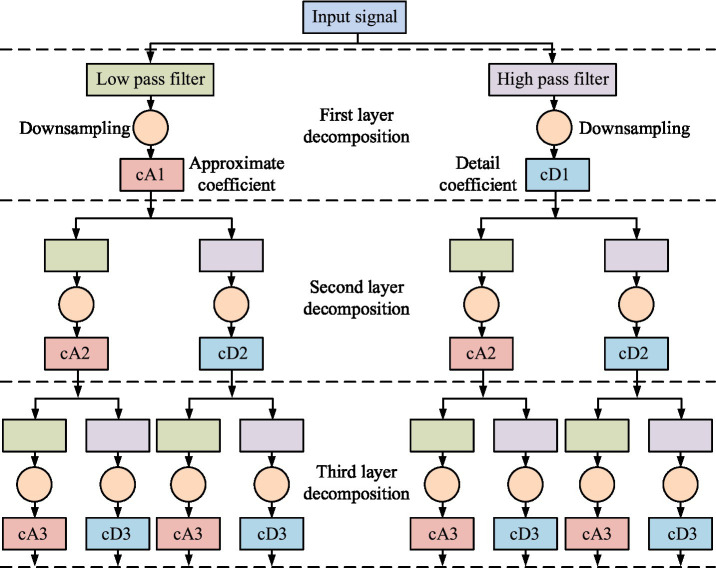
Schematic diagram of hierarchical decomposition of DWT algorithm.

In Equation 1, $cA_{j}$ and $cD_{j}$ are the approximation coefficient and detail coefficient of the *j*th layer, respectively, representing the low-frequency and high-frequency components of the signal; $\xi_{j,n}[q]$ and $\varsigma_{j,n}[q]$ respectively represent scale function and wavelet function; *n* and *q* represent time index and summation index, respectively. The hierarchical decomposition diagram of the DWT algorithm is shown in Figure 1.

In Figure 1, the input signal is first filtered through a low-pass filter and a high pass filter to extract low-frequency and high-frequency components, respectively. Then, downsampling is performed to generate approximation coefficients and detail coefficients for the first layer. The approximation coefficients continue to be decomposed layer by layer into the coefficients of the second and third layers through the same filtering and downsampling operations. This multi-level decomposition structure effectively captures the time-frequency characteristics of the signal. Low-frequency components represent the signal’s overall trend, while high-frequency components reflect details and noise. Further research is needed to extract TD, FD, and nonlinear features from the decomposed coefficients. Among them, the TDFE characterizes the overall trend and volatility of the signal by calculating the mean and variance, as shown in Equation 2.


$$\left\{ \begin{array}{l} \bar{M}=\frac{1}{O}\sum_{i=1}^{O}z_{i}\\ \sigma^{2}=\frac{1}{O}\sum_{i=1}^{O}(z_{i}-\bar{M}) \end{array}\right.$$ (2)

In Equation 2, $z_{i}$ denotes the *i*th sampling point of the signal; *O* represents the total number of sampling points of the signal; μ represents the arithmetic mean of the signal, reflecting the overall trend of the signal; $S^{2}$ represents a measure of signal dispersion and characterizes volatility. The extraction of FD features (FDFs) reflects the distribution of signals in the FD by calculating the spectral energy of each sub-band signal. The calculation of wavelet energy is shown in Equation 3 (24).


$$E_{j}=\sum_{n=1}^{N}|cD_{j}[n]\;|2$$ (3)

In Equation 3, $E_{j}$ denotes the detail coefficient energy of the *j*th layer. The calculation of the wavelet energy entropy is denoted in Equation 4.


$$H=-\sum_{j=1}^{J}\frac{E_{j}}{E_{\text{total}}}\log\frac{E_{j}}{E_{\text{total}}}$$ (4)

In Equation 4, *H* represents wavelet energy entropy; *J* denotes the total number of decomposition layers; $E_{\text{total}}$ denotes the total energy of all layers. For the extraction of nonlinear features, the complexity of capturing signals by calculating sample entropy is studied, and its expression is shown in Equation 5 (25).


$$\mathrm{SE}(a,b,N)=-\ln(G^{a}(b)/H^{a}(b))$$ (5)

**Figure 2:**
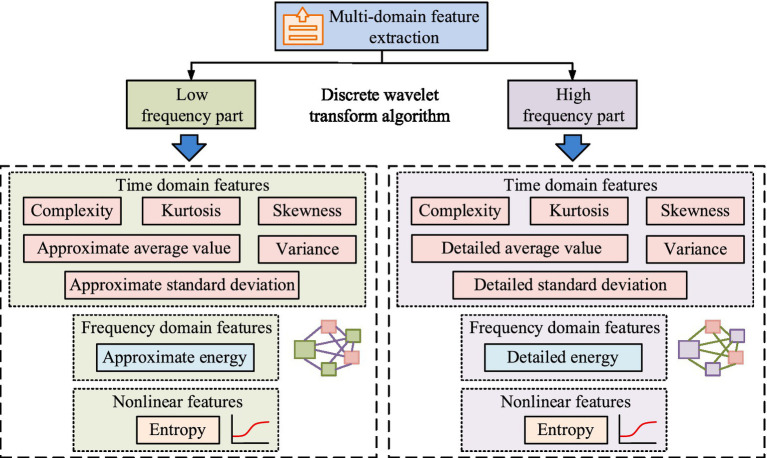
Structural diagram of MDFE based on DWT algorithm.

In Equation 5, SE(a,b,N) represents sample entropy; *a* denotes the embedding dimension; *b* denotes the similarity threshold; $G^{a}(b)$ denotes the amount of similar sequence pairs in the embedding dimension *a*; $H^{a}(b)$ represents the number of similar sequence pairs in the embedding dimension a+1 . Signal complexity refers to the irregularity or unpredictability of the health status signal constructed from multivariate health indicators. It is quantified using sample entropy, a non-linear feature extracted from the wavelet-decomposed signal. A higher sample entropy value indicates greater complexity in the health profile, which may reflect more varied or unstable health conditions requiring closer monitoring or more intensive care services. The structure diagram of MDFE based on DWT algorithm is shown in Figure 2.

In Figure 2, the DWT algorithm decomposes the input signal into low-frequency and high-frequency parts, corresponding to approximation coefficients and detail coefficients, respectively. The extracted features include TD features (TDFs), FDFs, and nonlinear features. The low-frequency part extracts TDFs, including complexity, kurtosis, skewness, approximate mean, variance, and approximate standard deviation, while the FDFs include approximate energy. The high-frequency part also extracts TDFs, including complexity, kurtosis, skewness, detailed mean, variance, and detailed standard deviation. FDFs extract detailed energy, and nonlinear features are mainly entropy. To quantify the explanatory power of features on classification targets, the study uses the information gain ratio to evaluate the importance of features. This index considers both the information entropy and conditional entropy of the features themselves, and its calculation is shown in Equation 6.


$$\mathrm{IGR}(F)=\frac{-\sum_{y\in R}g(r)\log g(r)-\sum_{f\in F}g(f)\sum_{y\in R}g(f,y)\log g(f,r)}{H(F)}$$ (6)

In Equation 6, IGR(F) means the information gain ratio; H(F) represents the entropy of feature *F*; *F* represents the feature variable to be evaluated; *R* represents the target variable; g(r) represents the edge probability of the target quantity *R* taking on the value *r*; g(f) represents the edge probability of feature *F* with a value of *f*; g(f,r) represents the joint probability of feature *F* being *f* and target quantity *R* being *r*. The study further introduces the non-linear statistical dependency relationship between mutual information capture features and target variables, which measures feature correlation by the difference between joint probability distribution and marginal probability distribution. The specific expression is shown in Equation 7 (26).


$$\mathrm{MI}(F,R)=\sum_{y\in R}\sum_{f\in F}g(f,r)\log\frac{g(f,r)}{g(f)p(r)}$$ (7)

In Equation 7, MI(F,R) refers to mutual information, measuring the statistical dependency between *F* and *R*. To achieve more accurate feature selection, a dynamic feature selection mechanism based on AST is studied and designed. AST uses the adjustment coefficient to balance the screening strictness by analyzing the distribution characteristics of feature importance, ensuring that significant related features are retained while controlling the feature dimension. The screening conditions are shown in Equation 8 (27).


$$\left\{ \begin{array}{l} \mathrm{IGR}(F)>\alpha \cdot \mathrm{IG}R_{\max}\\ \mathrm{MI}(F,Y)>\beta \cdot MI_{\max} \end{array}\right.$$ (8)

In Equation 8, α and β respectively represent the information gain ratio and the threshold coefficient of mutual information; $\mathrm{IG}R_{\max}$ and $MI_{\max}$ respectively represent the maximum information gain ratio and mutual information among all features. Finally, the RF model is constructed based on the selected feature subset, and the classification performance is improved through a weighted voting mechanism, as shown in Equation 9.


$$\left\{ \begin{array}{l} w_{t}=(A_{t})/\sum_{t=1}^{T}A_{t}\\ P(y=c)=\sum_{t=1}^{T}w_{t}\cdot I(\mathrm{ht}(x)=c) \end{array}\right.$$ (9)

**Figure 3:**
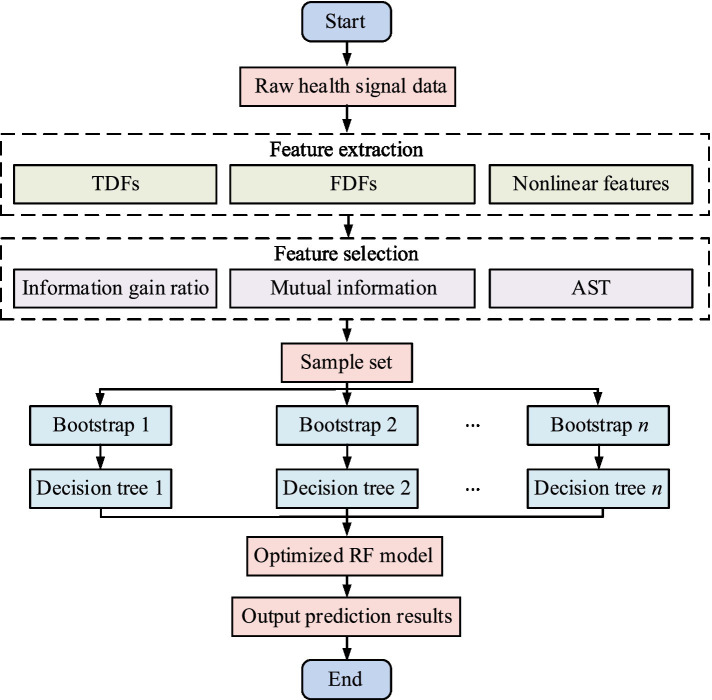
The flowchart of ORF.

In Equation 9, $w_{t}$ and $A_{t}$ respectively represent the weight and classification ACC of the *_t_* th decision tree; *T* represents the total amount of decision trees; P(y=c) means the probability of the model predicting the output category *c*; I(ht(x)=c) stands for indicator function. The schematic diagram of the ORF process is denoted in Figure 3.

In Figure 3, the ORF process starts from the raw health signal data and first performs feature extraction, including TD, FD, and nonlinear features. Next, feature selection is performed through information gain ratio, mutual information, and AST strategy. Then, the sample set for multiple self sampling is utilized to generate multiple decision trees. Finally, these decision trees are integrated to form an optimized RF model and the predicted results are output.

### 2.2 Construction of nursing service demand prediction model combining improved RF algorithm and logistic regression

The study optimizes the feature selection ability of RF through DWT and MDFE, effectively solving the redundancy and noise problems in high-dimensional data. However, the RF model has weak interpretability when dealing with linear relationships. Due to its interpretability and probabilistic output advantages in handling linear classification problems, LR has become an ideal choice to compensate for the shortcomings of RF (28). Therefore, a nursing service demand prediction model combining ORF algorithm and LR is developed, aiming to further improve the ACC and interpretability of older adult nursing service demand prediction by combining the nonlinear feature selection ability of RF and the linear modeling advantage of LR. The ORF algorithm excels in handling high-dimensional, non-linear relationships through its ensemble structure and robust feature selection capability. However, it lacks interpretability and may not efficiently capture linear dependencies. LR provides clear probabilistic outputs and model interpretability through feature coefficients, making it suitable for linear decision boundaries. By integrating ORF for feature selection and non-linear pattern capture and LR for linear modeling and interpretability, the hybrid model achieves both high predictive ACC and transparency.

Computationally, the ORF algorithm performs adaptive feature selection using mutual information and information gain ratio, reducing dimensionality and mitigating overfitting. The selected features are then fed into the LR model, which is optimized via batch gradient descent to minimize cross-entropy loss. Statistically, the hybrid approach benefits from the bias-variance trade-off: RF reduces variance through bagging, while LR provides low-bias linear estimates. The final prediction is a weighted combination of both models,ensuring a balance between non-linear and linear influences.

The study first passes the key feature subset selected by the ORF algorithm to the LR model. The selected feature matrix is set as $X\in R^{n\times m}$ , where *n* and *m* are the amount of samples and features, respectively, and the target variable is y∈{0,1} . The output of the LR model is denoted in Equation 10.


$$P(y=1|X)=\frac{1}{1+e^{-(\beta_{0}+\sum_{u=1}^{m}\beta_{u}x_{u})}}$$ (10)

**Figure 4:**
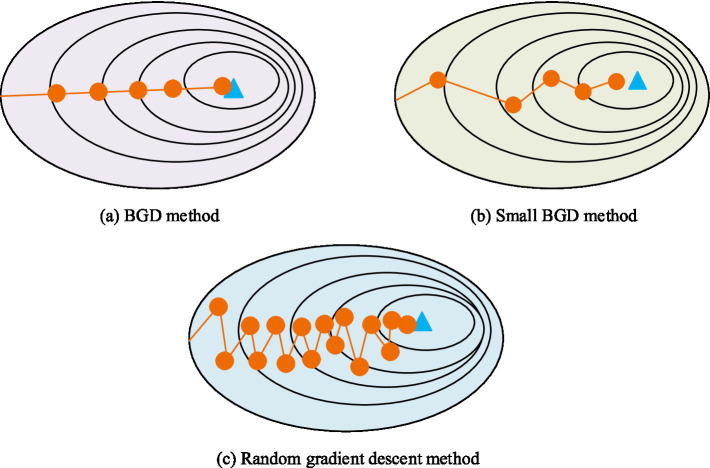
Schematic diagram of three GDMs. **(a)** BGD method; **(b)** small BGD method; **(c)** random gradient descent method.

In Equation 10, P(y=1∣X) represents the probability prediction output of the LR model; **X** represents the feature matrix; $b_{0}$ represents intercept term; $b_{u}$ refers to the coefficient of the *u*th feature; $x_{u}$ stands for the *u*th feature in the feature matrix. During the LR model’s parameter optimization process, the gradient descent algorithm can reduce the loss function (LF) by iteratively updating the model parameters (29). The gradient descent method (GDM) includes three methods: batch gradient descent (BGD), small BGD, and random gradient descent, as denoted in Figure 4.

Figure 4a shows the BGD method, which uses all training data to calculate gradients and update model parameters for each iteration. Its characteristic is convergence stability, but it has high computational overhead and is suitable for small-scale datasets. Figure 4b shows the small BGD method, which randomly selects a small batch of samples for each iteration to calculate the gradient, balancing computational efficiency and convergence stability. It is a commonly used optimization method in deep learning. Figure 4c shows the stochastic GDM, which uses only a single sample to update parameters in each iteration, resulting in fast computation speed but large gradient fluctuations, which may lead to unstable convergence. Due to the ability of BGD to provide more accurate gradient information, it helps the model better learn the global features of the data and improve the probability of converging to the global optimal solution. Therefore, the study uses BGD to optimize the LF of LR, to measure the difference between the predicted results of the model and the actual labels. The cross entropy LF is shown in Equation 11 (30).


$$\frac{\partial }{\partial \theta_{q}}Q(\theta )=\frac{1}{m}\sum_{\nu =1}^{m}(h_{\theta }(x^{\left(\nu \right)})-y^{\left(\nu \right)})x_{q}^{\left(\nu \right)}$$ (11)

In Equation 11, Q(θ) represents the cross entropy LF; q represents model parameters; $q_{q}$ represents the *_q_* th element in the model parameter vector q ; *_m_* denotes the total number of samples; $x^{\left(v\right)}$ and $y^{\left(v\right)}$ respectively represent the feature vector and true label of the *_v_* th sample; $h_{q}(x^{\left(v\right)})$ means the predicted value of the model; $\frac{\partial }{\partial \theta_{q}}$ represents the partial derivative of the LF parameters. To reduce the LF, the GDM is employed to update the model parameters, and the update rule is denoted in Equation 12.


$$\theta_{q+1}=q_{q}-a\frac{1}{m}\sum_{v=1}^{m}(h_{\theta }(x^{\left(v\right)})-y^{\left(v\right)})x_{q}^{\left(v\right)}$$ (12)

**Figure 5:**
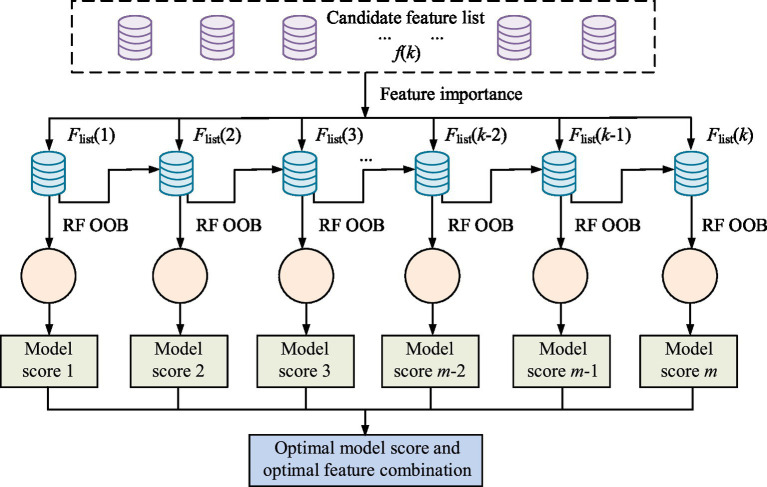
Schematic diagram of hybrid feature selection based on ORF algorithm and OOB.

In Equation 12, $\theta_{q+1}$ represents the *q*th updated parameter value; *a* stands for learning rate. To raise the generalization ability of the model, an improved RF algorithm is employed to select mixed features, and Out-of-Bag (OOB) is employed to verify and exam the effectiveness of the model (31). The schematic diagram of mixed feature selection based on ORF algorithm and OOB is shown in Figure 5.

In Figure 5, the process of mixed feature selection first calculates the importance of each feature in the candidate feature list, and verifies multiple feature subsets through an improved RF algorithm and OOB to obtain corresponding model scores. The final selection of the feature combination corresponding to the optimal model score aims to enhance the ACC of feature selection and the generalization ability of the model, thereby optimizing the overall predictive performance. The study validates the feature selection results using OOB estimation of RF to evaluate the predictive performance of a single decision tree. The calculation of OOB error is shown in Equation 13 (32).


$$\mathrm{OB}B_{t}=\frac{1}{|D_{\mathrm{oob}}|}\sum_{(x,y)=D_{\mathrm{oob}}}I(h_{t}(x)\neq y)$$ (13)

In Equation 13, $\mathrm{OB}B_{t}$ represents the OOB error of the t th tree; $|D_{\mathrm{oob}}|$ represents the size of the sample set $D_{\mathrm{oob}}$ outside the bag; $D_{\mathrm{oob}}$ represents the sample set outside the bag; $h_{t}(x)$ means the prediction result of the t th tree for sample x . To quantify the importance of features, the study calculates the change in error by replacing feature values, as denoted in Equation 14.


$$I(F_{i})=\frac{1}{T}\sum_{t=1}^{T}(\mathrm{OB}B_{t}^{p}-\mathrm{OB}B_{t})$$ (14)

In Equation 14, $I(F_{i})$ refers to the importance measure of feature $F_{i}$ ; $\mathrm{OB}B_{t}^{p}$ denotes the OOB error of the t th tree after feature $F_{i}$ permutation. The final research combines the feature importance of ORF and the coefficient of LR to construct the final prediction model, and its weighted prediction probability is shown in Equation 15.


$$P_{\text{final}}(y=1|X)=gP_{\mathrm{RF}}(y=1|X)+(1-g)P_{\mathrm{LR}}(y=1|X)$$ (15)

**Figure 6:**
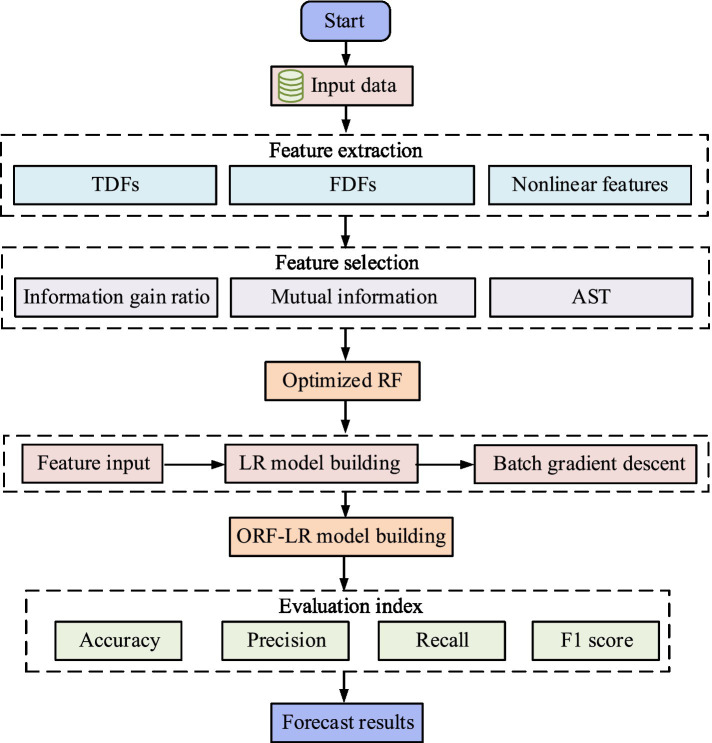
The flowchart of nursing service demand prediction combining improved RF and logistic regression.

In Equation 15, $P_{\text{ftnal}}(y=1|X)$ means the prediction probability of the target variable y taking a value of 1 when the final model is given the feature X ; g represents the weight coefficient; $P_{\mathrm{RF}}(y=1|X)$ and $P_{\mathrm{LR}}(y=1|X)$ represent the prediction probabilities of the RF model and LR model, respectively. The flowchart for predicting nursing service demand by combining ORF and LR is shown in Figure 6.

In Figure 6, the process of predicting the DOACS first extracts multi-domain features from the input data, and selects features through information gain ratio, mutual information, and AST. ORF selects key features and inputs them into the LR model to study the training of LR model parameters using BGD method. Finally, by weighting and integrating the prediction results of RF and LR, a comprehensive prediction probability is generated and the performance of the prediction model is assessed.

## 3 Results

This section first validates the performance of the ORF algorithm, and then analyzes the predictive performance and effectiveness of the older adult care service demand prediction model combining ORF and LR.

### 3.1 Performance verification of improved RF algorithm based on feature selection and AST

To prove the predictive performance of the improved RF algorithm in the DOACS, experiments were conducted on two public datasets, the Medical Endurance Panel Survey (MEPS) and the National Health and Aging Trends Study (NHATS). Among them, the MEPS dataset is managed by the Healthcare Research and Quality Agency under the US Department of Health and Human Services, covering medical expenses, disease history, and nursing service usage records of 8,500 older adult people. The NHATS dataset is funded by the National Institute on Aging in the United States and implemented by the Bloomberg School of Public Health at Johns Hopkins University. It contains multidimensional health data of 12,000 older adult individuals, including physiological, psychological, and social support data. This study utilized two large-scale public datasets, MEPS (N = 8,500) and NHATS (N = 12,000), providing sufficient sample sizes. Model performance was evaluated using 10-fold cross-validation to ensure robust results. Comparisons of metrics such as ACC and recall (Rec) between models were supported by statistical tests, and 95% confidence intervals were calculated to verify the significance of performance differences. The experimental environment and parameter settings are denoted in Table 1.

**Table 1:** Experimental environments and parameter settings.

Experimental environments		Parameter settings	
Name	Types	Parameters	Values
Processor	Intel Core i7-10700K @ 3.80GHz	Number of decision trees	500
Memory	32GB DDR4 3,200 MHz	Maximum depth	10
Graphics card	NVIDIA GeForce RTX 3060	Minimum number of leaf samples	5
Storage	1 TB NVMe SSD	Learning rate	0.01
Operating system	Windows 10	Iterations	500
Programming language	Python 3.8	Batch size	32

**Figure 7:**
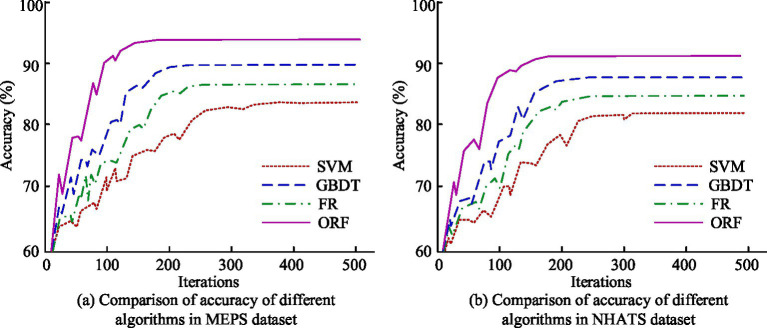
Comparison of ACC of different algorithms in MEPS and NHATS datasets. **(a)** Comparison of accuracy of different algorithms in MEPS dataset. **(b)** Comparison of accuracy of different algorithms in NHATS dataset.

The study first compared the ACC of the ORF algorithm in classifying older adult care service needs in the MEPS and NHATS datasets, and compared it with SVM, Gradient Boosted Decision Tree (GBDT), and traditional RF algorithm to exam the reliability of the proposed algorithm. The findings are denoted in Figure 7. In Figure 7a, under the MEPS dataset, when the number of iterations was 100, the ACC of SVM, RF, and GBDT were 71.02, 73.89, and 78.23%, respectively, and the ACC of ORF was 90.26%. When the amount of iterations reached 300, the ACC of the four algorithms was 83.11, 86.78, 89.25, and 93.24%, respectively. In Figure 7b, under the NHATS dataset, with 100 iterations, the ACC of the four algorithms was 68.27, 71.06, 74.63, and 87.15%, respectively. When the amount of iterations reached 300, the ACC increased to 81.68, 84.23, 88.12, and 91.36%, respectively. The findings denoted that the ORF algorithm had high ACC in both datasets, outperforming other compared algorithms.

**Figure 8:**
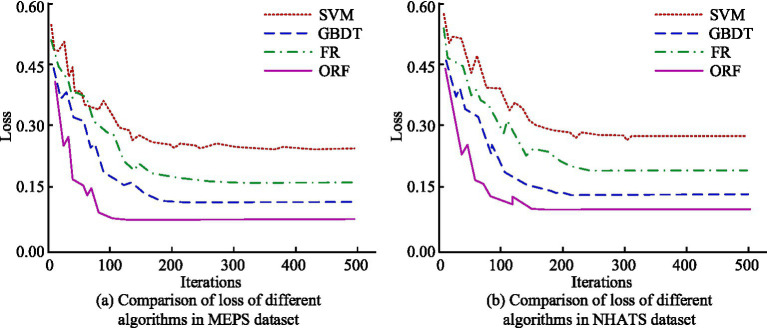
Comparison of loss between different algorithms in MEPS and NHATS datasets. **(a)** Comparison of loss of different algorithms in MEPS dataset. **(b)** Comparison of loss of different algorithms in NHATS dataset.

To validate the convergence performance of the ORF algorithm, the loss values were analyzed on the MEPS and NHATS datasets and compared with traditional algorithms. The outcomes are denoted in Figure 8. In Figure 8a, under the MEPS dataset, when the number of iterations was 100, the losses of SVM, RF, GBDT, and the proposed algorithm were 0.328, 0.276, 0.184, and 0.082, respectively. When the amount of iterations increased to 300, the losses of the four algorithms were 0.243, 0.167, 0.121, and 0.078, respectively. In Figure 8b, under the NHATS dataset, when the number of iterations was 100, the losses of SVM, RF, and GBDT were 0.386, 0.324, and 0.243, respectively, and the loss of the developed algorithm was 0.128. When the amount of iterations reached 300, the losses of the four algorithms were 0.282, 0.201, 0.142, and 0.115, respectively. The findings denoted that the ORF algorithm had better convergence speed than other compared algorithms in both datasets, verifying the feasibility of the algorithm.

**Figure 9:**
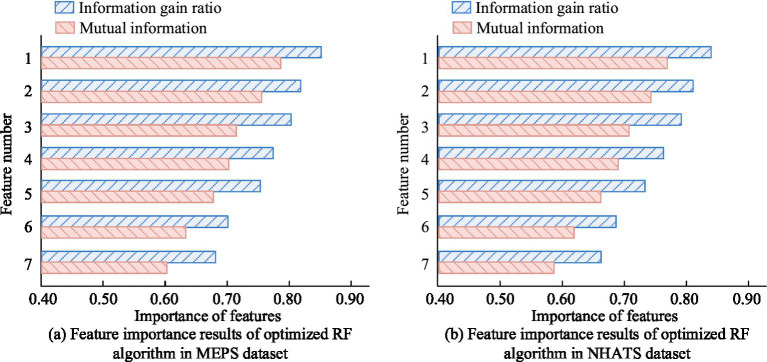
Feature importance results of optimized RF algorithm in MEPS and NHATS datasets. **(a)** Feature importance results of optimized RF algorithm in MEPS dataset. **(b)** Feature importance results of optimized RF algorithm in NHATS dataset.

Further analysis was conducted on the feature importance ranking of the ORF algorithm in the MEPS and NHATS datasets. The main features included the number of chronic diseases, daily activity ability scores, mental health scores, medical expenses, social support scores, hospitalization frequency, and medication frequency, numbered 1–7, respectively. The indicators were information gain ratio and mutual information, as shown in Figure 9. In Figure 9a, the information gain ratios of the seven main features in the MEPS dataset were 0.85, 0.82, 0.80, 0.78, 0.75, 0.70, and 0.68, respectively, and the mutual information was 0.78, 0.75, 0.72, 0.70, 0.68, 0.63, and 0.60, respectively. In Figure 9b, in the NHATS dataset, the information gain ratios of the seven main features were 0.84, 0.81, 0.79, 0.76, 0.73, 0.69, and 0.66, respectively, and the mutual information was 0.77, 0.74, 0.71, 0.69, 0.66, 0.62, and 0.59, respectively. The findings denoted that in both datasets, the number of chronic diseases and daily activity ability scores were the two most important features for predicting the DOACS. By optimizing the RF algorithm, the most critical features for predicting the DOACS could be effectively identified. Features such as number of chronic diseases and daily activity ability scores were identified as the most influential in predicting older adult care service demand. This is because these features directly reflect the level of functional dependency and medical need, which are primary drivers of care resource utilization. For instance, a higher number of chronic conditions often correlates with increased need for medical monitoring and assistance, while lower activity scores indicate greater dependency in daily living, necessitating more intensive care services.

**Figure 10:**
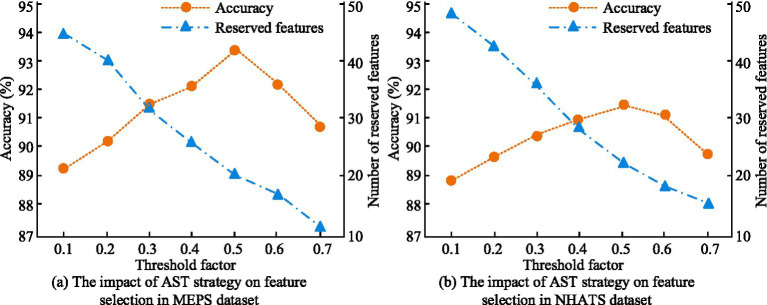
Impact of AST strategy on feature selection in MEPS and NHATS datasets. **(a)** The impact of AST strategy on feature selection in MEPS dataset. **(b)** The impact of AST strategy on feature selection in NHATS dataset.

To investigate the impact of AST strategy on feature selection, an analysis was conducted on the number of retained features and ACC under different threshold coefficients in the MEPS and NHATS datasets. The findings are denoted in Figure 10. In Figure 10a, in the MEPS dataset, when the threshold coefficient was 0.5, the ACC of the AST strategy for feature selection was 93.24%, and the number of retained features was 20. When the threshold coefficient increased to 0.7, its ACC and number of retained features were 90.67% and 12, respectively. In Figure 10b, in the NHATS dataset, when the threshold coefficient as 0.5, the AST strategy had the highest ACC in feature selection, at 91.36%, with 22 retained features. When the threshold coefficient reached 0.7, the ACC was 89.78% and the number of retained features was 15. The results indicated that by adjusting the threshold coefficient of the AST strategy reasonably, the feature dimension could be reduced while retaining key features, thereby raising the predictive performance of the model.

To further validate the ORF algorithm’s superiority, an analysis was carried out on its ACC, Rec, and F1 score (F1) in two datasets, and compared with other algorithms. The findings are denoted in Table 2. In Table 2, the proposed algorithm outperformed SVM, GBDT, and RF algorithms in terms of ACC, Rec, and F1 in the MEPS dataset, with values of 0.926, 0.915, and 0.920, respectively. In the NHATS dataset, the proposed algorithm also performed the best, with an ACC of 0.908, a Rec of 0.895, and an F1 of 0.901, surpassing other algorithms. This indicated that the OURS algorithm had better predictive performance on both datasets, especially in improving Rec and F1, demonstrating its excellence in predicting the DOACS.

**Table 2:** Performance comparison of different algorithms in MEPS and NHATS datasets.

Datasets	Algorithms	ACC	Rec	F1
MEPS	SVM	0.815	0.802	0.808
	GBDT	0.887	0.873	0.880
	RF	0.859	0.846	0.852
	ORF	0.926	0.915	0.920
NHATS	SVM	0.801	0.793	0.797
	GBDT	0.875	0.862	0.868
	RF	0.836	0.821	0.828
	ORF	0.908	0.895	0.901

### 3.2 Analysis of prediction results for older adult care service demand

**Figure 11:**
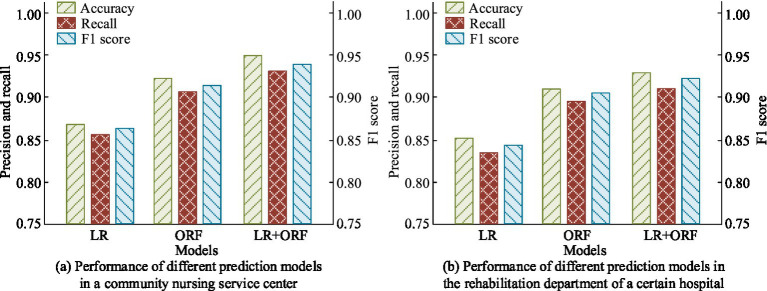
Impact of AST strategy on feature selection in MEPS and NHATS datasets. **(a)** Performance of different prediction models in a community nursing service center. **(b)** Performance of different prediction models in the rehabilitation department of a certain hospital.

To exam the actual prediction effectiveness of the prediction model combining ORF algorithm and LR, the ACC, Rec rate, and F1 of the prediction were analyzed in two actual scenarios: a community nursing service center and a rehabilitation department of a hospital. The results were compared with traditional LR and ORF models, as shown in Figure 11. In Figure 11a, in the community nursing service center scenario, the ACC of traditional LR and ORF models were 0.872 and 0.932, respectively, with Rec rates of 0.852 and 0.916, and F1s of 0.862 and 0.924, respectively. The ACC, Rec, and F1 of the proposed prediction model were 0.953, 0.926, and 0.939, respectively. In Figure 11b, in the hospital rehabilitation scene, the ACC of the three prediction models was 0.872, 0.914, and 0.935, the Rec rates were 0.835, 0.897, and 0.912, and the F1s were 0.845, 0.905, and 0.923, respectively. The findings denoted that the proposed prediction model could effectively improve the ACC and reliability of predictions, and was suitable for multidimensional prediction of older adult care needs, providing more accurate support for resource allocation.

**Figure 12:**
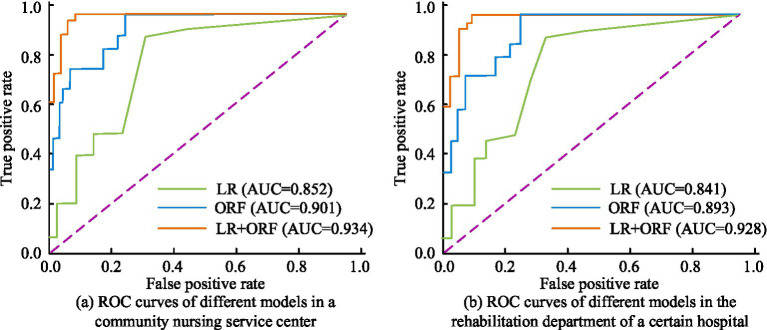
ROC curve results of prediction models in two actual scenarios. **(a)** ROC curves of different models in a community nursing service center. **(b)** ROC curves of different models in the rehabilitation department of a certain hospital.

The Receiver Operating Characteristic (ROC) curves of the raised prediction model were analyzed in two practical scenarios and compared with traditional LR and ORF models. The findings are denoted in Figure 12. In Figure 12a, in the community nursing service center scenario, the Area Under Curve (AUC) of the traditional LR and ORF were 0.852 and 0.901, respectively, and the AUC of the proposed model was 0.934. In Figure 12b, the AUC of the three models in the hospital rehabilitation scene were 0.841, 0.893, and 0.928, respectively. The findings demonstrated that the prediction model combining ORF algorithm and LR had the best performance in predicting the DOACS, demonstrating better discrimination ability and prediction ACC in two practical application scenarios.

This study conducted statistical tests on the ACC, Rec, F1, and AUC of the proposed model compared to the baseline ORF model, and analyzed the 95% confidence interval. The results are shown in Table 3. In Table 3, the 95% confidence intervals for the ACC, Rec, F1, and AUC indicators of the proposed model in the community nursing center scenario were [0.941, 0.965], [0.909, 0.943], [0.926, 0.952], and [0.919, 0.949], respectively, all of which are higher than the corresponding intervals of the ORF model. Statistical tests showed that, except for the Rec index with a *p*-value of 0.04, the *p*-values of the other three indicators were all less than 0.01, indicating that the performance improvement is statistically significant. In the context of hospital rehabilitation, the proposed model also performed well, with 95% confidence intervals for ACC, Rec, F1, and AUC of [0.922, 0.948], [0.894, 0.930], [0.909, 0.937], and [0.912, 0.944], respectively. The results showed that the hybrid model combined with LR not only achieved statistically significant improvements in all key evaluation indicators, but also maintained consistent performance gains in different application scenarios, verifying the effectiveness and robustness of the method.

**Table 3:** Statistical comparison and confidence intervals for model performance metrics.

Scenario	Metric	95% CI		*p*-value
		ORF+LR	ORF	
Community nursing center	Precision	[0.941, 0.965]	[0.918, 0.946]	< 0.01
	Recall	[0.909, 0.943]	[0.897, 0.935]	0.04
	F1-Score	[0.926, 0.952]	[0.909, 0.939]	< 0.01
	AUC	[0.919, 0.949]	[0.883, 0.919]	< 0.01
Hospital rehabilitation	Precision	[0.922, 0.948]	[0.899, 0.929]	0.01
	Recall	[0.894, 0.930]	[0.877, 0.917]	0.04
	F1-Score	[0.909, 0.937]	[0.889, 0.921]	0.01
	AUC	[0.912, 0.944]	[0.874, 0.912]	< 0.01

*p* < 0.01 indicates reaching a highly significant level.

**Figure 13:**
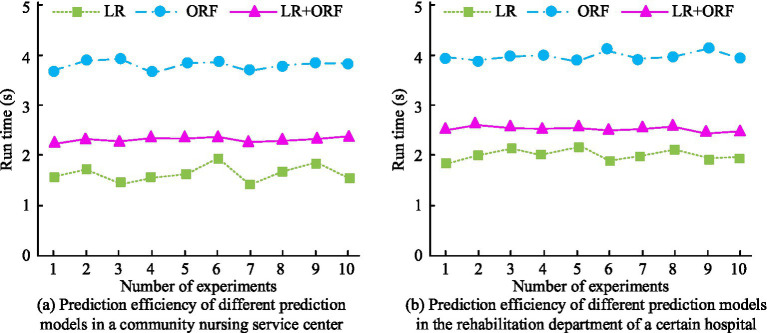
Prediction efficiency of different prediction models in two practical scenarios. **(a)** Prediction efficiency of different prediction models in a community nursing service center. **(b)** Prediction efficiency of different prediction models in the rehabilitation department of a certain hospital.

To explore the predictive efficiency of the raised prediction model, its running time was analyzed in two actual scenarios, and the findings are denoted in Figure 13. In Figure 13a, in the community nursing service center scenario, the average running time of the traditional LR model and ORF model were 1.62 s and 3.82 s, respectively. Compared with them, the running time of the raised model was 2.23 s, which was 41.62% shorter than that of the ORF model. In Figure 13b, the average running time of the three models in the hospital rehabilitation scene was 2.03 s, 3.98 s, and 2.54 s, respectively. Compared with the ORF model, the running time of the proposed model was reduced by 36.18%. The findings denoted that although the raised model may have better predictive performance than other models, its predictive efficiency was similar to the ORF model and slightly lower than the traditional LR model. The proposed model can be deployed in community health centers or hospital rehabilitation departments to proactively identify older adult individuals at high risk of requiring care services. By inputting routinely collected health indicators, the model outputs a probability of high service demand. This enables targeted resource allocation, personalized care planning, and early intervention, thereby improving efficiency and equity in older adult care provision.

## 4 Discussion

The study proposed a prediction model for older adult care services that combines ORF algorithm and LR, aiming to strengthen the ACC and reliability of predictions. The experimental findings denoted that on the two common datasets of MEPS and NHATS, the ACC of the ORF algorithm reached 90.26 and 87.15%, respectively, significantly better than traditional RF, SVM, and GBDT algorithms. The combination of improved ORF algorithm and LR prediction model showed better ACC, Rec, and F1 than traditional LR and RF models in practical scenarios of community nursing service centers and hospital rehabilitation departments. In the community nursing service center scenario, the ACC, Rec, and F1 of the proposed model were 0.953, 0.926, and 0.939, respectively, while the corresponding indicators of the traditional LR model and traditional RF model were 0.872, 0.852, 0.862, and 0.932, 0.916, and 0.924, respectively. Besides, the AUC of the proposed model reached 0.934 and 0.928 in both practical scenarios, demonstrating better discrimination ability and prediction ACC.

This study makes marked progress compared to prior work. Unlike the federated RF model in reference (6), which excels in data integration but overlooks feature selection and interpretability, our method integrates adaptive feature selection with LR to address both aspects. The model in reference (7) achieved lower ACC using RF alone, whereas our hybrid model yielded notable improvements in ACC and Rec. While studies like references (8, 9) optimized single algorithms for disease prediction, our fusion of ORF and LR produced stronger performance. Likewise, though LR studies references (13, 14) advanced linear classification and imbalance handling, they struggle with complex data.

Unlike prior studies that primarily focus on single-disease prediction, proposed model addresses the multidimensional and composite nature of older adult care service demand, which is influenced by a range of health, functional, and social factors. While studies such as Hauschild et al. (6) and Mbonyinshuti et al. (10) use RF for biomedical or drug demand prediction, they do not integrate interpretable linear modeling or adaptive feature selection in the context of older adult care. The propoesd model not only improves predictive ACC but also enhances model interpretability.

The proposed framework is generalizable beyond the older adult care context. It can be adapted to other domains requiring high-dimensional feature selection and interpretable prediction, such as mental health service planning, disability support assessment, or resource forecasting in public health. This expands the relevance of our work beyond the typical scope of disease-specific models.

A key limitation is our model’s reliance on high-quality data, which is vulnerable to gaps and noise in practice. Future efforts will incorporate images and speech to boost ACC further.

## 5 Conclusion

The research proposed a prediction model for older adult care service demand that combines ORF algorithm and LR. Through DWT MDFE and adaptive feature selection strategy, the feature processing ability was optimized, and the prediction ACC and interpretability of the model were improved. The experimental findings denoted that the model exhibited high ACC, Rec, and F1 in both MEPS and NHATS datasets, as well as in practical scenarios of community nursing service centers and hospital rehabilitation departments, significantly outperforming traditional methods. This study provides a new and effective tool for predicting the DOACS, which can provide strong decision support for resource allocation and service planning of older adult care service institutions and government departments.

## Data Availability

The original contributions presented in the study are included in the article/supplementary material, further inquiries can be directed to the corresponding author.
